# Supplementary material for: Does laryngeal reinnervation or type I thyroplasty give better voice results for patients with unilateral vocal fold paralysis (VOCALIST): study protocol for a feasibility randomised controlled trial
Source: BMJ Open. 2017 Sep 29;7(9):e016871. doi: 10.1136/bmjopen-2017-016871 (PMC5640104; doi:10.1136/bmjopen-2017-016871)
Supplement: Author's manuscript [file bmjopen-2017-016871.draft_revisions.pdf]

BMJ Open is committed to open peer review. As part of this commitment we make the peer review history of every article we publish publicly available.

When an article is published we post the peer reviewers' comments and the authors' responses online. We also post the versions of the paper that were used during peer review. These are the versions that the peer review comments apply to.

The versions of the paper that follow are the versions that were submitted during the peer review process. They are not the versions of record or the final published versions. They should not be cited or distributed as the published version of this manuscript.

BMJ Open is an open access journal and the full, final, typeset and author-corrected version of record of the manuscript is available on our site with no access controls, subscription charges or pay-per-view fees (http://bmjopen.bmj.com).

If you have any questions on BMJ Open's open peer review process please email editorial.bmjopen@bmj.com

BMJ Open

Does Laryngeal Reinnervation or Type I Thyroplasty give better voice results for patients with Unilateral Vocal Fold Paralysis (VOCALIST): study protocol for a feasibility randomised controlled trial

Journal:	*BMJ Open*
Manuscript ID	bmjopen-2017-016871
Article Type:	Protocol
Date Submitted by the Author:	15-Mar-2017
Complete List of Authors:	Blackshaw, Helen; University College London Ear Institute, evidENT<br>Carding, Paul; Australian Catholic University Faculty of Health Sciences<br>Jepson, Marcus; University of Bristol School of Social and Community Medicine<br>Mat Baki, Marina; Pusat Perubatan Universiti Kebangsaan Malaysia<br>Ambler, Gareth; University College London<br>Schilder, Anne; University College London Ear Institute, evidENT<br>Morris, Stephen; University College London, Department of Applied Health Research<br>Degun, Aneeka; University College London Ear Institute, evidENT<br>Yu, Rosamund; University College London Hospitals NHS Foundation Trust, Biomedical Research Centre<br>Husbands, Samantha; University of Bristol School of Social and Community Medicine<br>Knowles, Helen; University College London Hospitals NHS Foundation Trust, Royal National Throat, Nose and Ear Hospital<br>Walton, Chloe; Australian Catholic University Faculty of Health Sciences<br>Karagama, Yakubu; Central Manchester University Hospitals NHS Foundation Trust, Manchester Royal Infirmary<br>Heathcote, Kate; Poole Hospital NHS Foundation Trust<br>Birchall, Martin; University College London Ear Institute
**Primary Subject Heading**:	Ear, nose and throat/otolaryngology
Secondary Subject Heading:	Surgery
Keywords:	Adult otolaryngology < OTOLARYNGOLOGY, Laryngology < OTOLARYNGOLOGY, Speech pathology < OTOLARYNGOLOGY, Otolaryngology < SURGERY, Clinical trials < THERAPEUTICS

SCHOLARONE™
Manuscripts

Does Laryngeal Reinnervation or Type I Thyroplasty give better voice results for patients with Unilateral Vocal Fold Paralysis (VOCALIST): study protocol for a feasibility randomised controlled trial

Helen Blackshaw[1], Paul Carding[2], Marcus Jepson[3], Marina Mat Baki[4], Gareth Ambler[5], Anne Schilder[1], Stephen Morris[6], Aneeka Degun[1], Rosamund Yu[7], Samantha Husbands[3], Helen Knowles[8], Chloe Walton[2], Yakubu Karagama[9], Kate Heathcote[10] and Martin Birchall[1]

[1] Ear Institute, University College London, UK

[2] School of Allied Health, Australian National Catholic University, Australia

[3] School of Social and Community Medicine, University of Bristol, UK

[4] Faculty of Medicine, National University of Malaysia, Malaysia

[5] Statistical Science, University College London, UK

[6] Department of Applied Health Research, University of London, UK

[7] UCL Hospitals Biomedical Research Centre, University College London Hospitals NHS Foundation Trust, UK

[8] Royal National Throat, Nose and Ear Hospital, University College London Hospitals NHS Foundation Trust, UK

[9] Department of Otolaryngology-Head & Neck Surgery, Manchester Royal Infirmary, UK

[10] Department of Otolaryngology, Poole Hospital, UK

Corresponding author:

Dr Helen Blackshaw

Ear Institute, University College London, Royal National Throat, Nose and Ear Hospital, 330 Grays Inn Road, London WC1X 8DA

h.blackshaw@ucl.ac.uk

020 3108 9326

Word count (excluding title page, abstract, figures and references): 3525 words

For peer review only

For peer review only - http://bmjopen.bmj.com/site/about/guidelines.xhtml

ABSTRACT

Introduction: A functioning voice is essential for normal human communication. A good voice requires two moving vocal folds; if one fold is paralysed (unilateral vocal fold paralysis-UVFP) people suffer from a breathy, weak voice that tires easily and is unable to function normally. UVFP can also result in choking and breathlessness. Current treatment for adults with UVFP is speech therapy to stimulate recovery of vocal fold (VF) motion or function and/or injection of the paralysed VF with a material to move it into a more favourable position for the functioning VF to close against it. When these therapies are unsuccessful, or only provide temporary relief, surgery is offered. The two available surgical techniques are; i) placing a synthetic implant next to the fold (type I thyroplasty), or ii) the newer approach of re-supplying the nerve that supplies the fold (non-selective laryngeal reinnervation). Currently there is no robust evidence to determine which is the best surgical option for adults with UVFP.

Methods and analysis: A feasibility study to test the practicality of running a multi-centre, randomised clinical trial of surgery for UVFP, including; a) a qualitative study to understand the recruitment process and how it operates in clinical centres; and b) a small randomised trial of 30 participants recruited at 3 UK sites comparing laryngeal reinnervation to thyroplasty. Participants will be followed up for 12 months. The primary outcome focuses on recruitment and retention, with the secondary outcomes covering voice, swallowing and quality of life.

Ethics and dissemination: Ethical approval was received from NRES - Committee Bromley (reference number 11/LO/0583). In addition to dissemination of results through presentation at scientific conferences and publication of peer-reviewed articles, results will be shared with the key clinician and patient groups required to develop the future large scale RCT.

Trial registration: ISRCTN90201732; registered on 16 December 2015.

For peer review only

For peer review only - http://bmjopen.bmj.com/site/about/guidelines.xhtml

STRENGTHS AND LIMITATIONS OF THIS STUDY

- This is the first clinical trial to be run in the UK to compare the effectiveness of two surgical approaches for restoration of laryngeal function in adults with Unilateral Vocal Fold Paralysis (UVFP)
- This study has been developed in response to; a) patient demand and b) a systematic review that reported a low quality of current literature available on laryngeal reinnervation and proposed that a formal prospective trial was required using standardised, internationally accepted outcomes.
- A similar trial, comparing laryngeal reinnervation and type I thyroplasty in adults with UVFP, was previously attempted in the US, but was suspended prematurely due to irregularity in obtaining inform consent and low accrual. We acknowledge the challenges associated with recruiting to a phase III surgical study of this type and have sought to overcome them by; a) learning from this previous trial; b) designing this rigorous feasibility study to be conducted prior to running a larger scale phase III RCT; and c) involving UVFP patients throughout the feasibility study design, set up, analysis and dissemination processes.
- Our research team is multi-disciplinary and our study design incorporates both qualitative and quantitative components.
- Whilst our study focuses on voice-related criteria for inclusion, our design includes outcomes relating to both the voice and swallowing.
- Although participants and study personnel are not blinded to their allocated treatment group, the speech therapist and neurophysiologist outcome raters are blinded.
- Very few ENT surgeons in the UK are trained to perform laryngeal reinnervation and so our study is limited to recruiting at those relevant UK sites.
- The short follow-up period for this study (6 months for all participants and 12 months where possible) is a limitation due to budget constraints. However, all patients are followed up clinically in the long-term.

For peer review only

For peer review only - http://bmjopen.bmj.com/site/about/guidelines.xhtml

INTRODUCTION

The larynx provides the main tool for human social interaction. The vocal folds are innervated by the recurrent laryngeal nerve (RLN) and when this nerve supply to one vocal fold is interrupted, unilateral vocal fold paralysis (UVFP) ensues. Patients suffering with UVFP typically experience a hoarse, weak voice [1] that tires easily, with some also suffering from breathing and swallowing problems. Quality of life of these patients is significantly reduced and social and employment opportunities are compromised [2 3] .Therefore, optimum rehabilitation of the paralysed larynx is important for health and wealth alike.

The aim of treatment for UVFP is to achieve optimum vocal fold (VF) closure for voice production. Where speech therapy alone is insufficient to improve the voice, surgical treatment is undertaken. Whilst short-term relief of symptoms can be achieved via injection of the paralysed VF with a material to improve its position for the functioning VF to close against it, permanent results are only possible by two different operations: type I thyroplasty or non-selective laryngeal reinnervation. The former uses a synthetic implant to move the paralysed VF into a better position for speaking, whilst the latter re-supplies nerves to improve muscle bulk and tone. Thyroplasty is a one-hour operation under local anaesthetic where the medium grade silastic implant is inserted through a window in the cartilage lamina of the larynx. This implant medialises the paralysed vocal fold in the midline position allowing the normal opposite vocal fold to make firm contact and produce a stronger voice. However, voice improvement may be short-term due to progressive atrophy of the denervated laryngeal muscles [4]. Furthermore, this procedure does not restore vocal fold tension, meaning that pitch variation remains suboptimal [5 6]. Reports on short- and long-term voice outcomes of patients with thyroplasty are sparse and methodologically poor [7]. The newer approach to restoring laryngeal function in UVFP patients, reinnervation, is a two-hour operation under general anaesthesia in which the injured RLN and the donor nerve are identified and anastomosed. Its aim is to restore innervation to the larynx by 'borrowing' the activity of other motor nerves, specifically the ansa cervicalis. As such it re-establishes the tone and bulk of the vocal folds during speech and re-enables pitch control resulting in normal or near normal voice. The operation is safe and results in sustained improvement of glottal closure, maintenance of the vocal fold edge and significant improvements in voice quality have been reported [8].

A systematic review of studies on laryngeal reinnervation revealed that the quality of current literature is low and a formal prospective trial was recommended using standardised, internationally accepted outcomes [9]. Before embarking on a trial that will use public resources and the time of patients and healthcare staff, we need to confirm that running a trial is feasible and that the chosen outcome measures truly reflect voice quality and patients' perceptions. For these reasons, and others given above, a feasibility study is critical prior to designing, with patients, a multi-centre, phase III randomised clinical trial of treatment for this disabling condition.

For peer review only

For peer review only - http://bmjopen.bmj.com/site/about/guidelines.xhtml

METHODS AND ANALYSIS

Objectives

The primary research objective is to determine the feasibility of performing a multi-centre phase III randomised controlled trial of laryngeal reinnervation versus type I thyroplasty for adult patients with UVFP.

The secondary research objectives are to test the feasibility of:

1) The multicentre recruitment process, including continuous improvement to the process based on qualitative methodology.
2) Recruiters being able to present true equipoise with the treatment arms.
3) The randomisation processes and to investigate reasons for any difficulties that affect recruitment.
4) The utility of the following characteristics of the proposed primary and secondary outcomes: variability across patients, variability over time, differences in outcome between randomised groups over time.
5) Process of follow up visits.
6) Means of gathering health economics and health-related quality of life data suitable for measuring cost-effectiveness.

Study design

The feasibility study will consist of two research approaches run in parallel;

1) Qualitative: The QuinteT Recruitment Intervention (QRI) to understand the recruitment process and how it operates in clinical centres, so that sources of recruitment difficulties can be identified and suggestions made to change aspects of design, conduct, organisation or training that could then lead to improvements in recruitment [10].

2) Quantitative: A small randomised surgical trial of 30 patients to compare laryngeal reinnervation to thyroplasty. Patients will be randomised in a 1:1 ratio.

The study is single blinded whereby the raters of the secondary quantitative outcome of acoustic measures will be blinded to the timepoint of the data recordings (preoperative or postoperative) and blinded to surgical intervention (laryngeal reinnervation or type I thyroplasty).

Participants

Inclusion criteria

1) UVFP due to unilateral recurrent laryngeal nerve paralysis of traumatic, iatrogenic or idiopathic origin of between 6 and 60 months duration. Or symptoms that have not sufficiently improved with speech therapy alone, as determined by the patient and agreed by a multidisciplinary clinical team, after 6 months and pending a surgical decision.

For peer review only

2) Age from 18 to 70 years old.
3) Male or female.
4) Able to provide informed consent.
5) A significant voice disorder as measured by perceptual rating (GRBAS Grade ≥2)[11] and Voice Handicap Index (VHI-10 score >16) [12].
6) Common laryngeal electromyography (LEMG, neurophysiological) criteria (Koufman Grades 2-5)[13] in either the thyroarytenoid (TA) or posterior cricoarytenoid (PCA) muscle on the paralysed side.

Exclusion criteria

1) Impaired vocal fold mobility but a normal EMG (Koufman Grade I).
2) Severe lung disorders.
3) Structural vocal fold lesions such as polyp.
4) Previous laryngeal framework surgery.
5) Cricoarytenoid joint fixation (CAJF).
6) Significant non-laryngeal speech abnormality (severe dysarthria determined by a panel of trained speech therapists).
7) Previous Level 2, 3 or 4 thyroid neck dissection.
8) Previous ipsilateral surgical neck dissection.
9) Previous radiotherapy to the head and neck.
10) Laryngeal injection of a rapidly absorbable material in the last 6 months.
11) Previous laryngeal injection of a non-rapidly absorbable material (e.g. bioplastics, VOX).
12) Neuromuscular disease affecting the larynx or multiple cranial nerve palsies.

Setting

The study is Sponsored by University College London Hospitals NHS Foundation Trust. Potential participants will be recruited from three UK hospitals; the Royal National Throat, Nose and Ear Hospital (University College London Hospitals NHS Foundation Trust), Manchester Royal Infirmary (Central Manchester University Hospitals NHS Foundation Trust) and Poole Hospital (Poole Hospital NHS Foundation Trust). The study is run through the University College London evidENT team, with qualitative data analysed by the University of Bristol and quantitative data analysed by University College London and Australian Catholic University.

Interventions

Laryngeal Reinnervation: The surgical technique is an unselective reinnervation [14] that aims to improve the muscle tone and bulk. Performed under general anaesthesia, the injured RLN and the donor nerve are identified and anastomosed.
Type 1 Thyroplasty: This medialisation/augmentation technique is a static technique [15], performed under local anaesthesia that aims to improve the positioning of the paralysed vocal fold. It uses a medium grade silastic implant readily available in different sizes according to size of larynx and

For peer review only - http://bmjopen.bmj.com/site/about/guidelines.xhtml

gender of the patient. The correct size can be determined intraoperatively by using a measuring device while listening and visualising the larynx with flexible fibreoptic scope simultaneously.

Outcome measures

The primary outcomes are, from those patients who were eligible to enter the study:

1) Whether the patient was randomised (yes/no).
2) Whether the participant successfully received the allocated operation (yes/no).
3) Whether the participant completed the trial (yes/no).

The quantitative secondary outcomes are:

1) General health status measured using the validated EQ-5D-5L [16] questionnaire. Participants will self-administer the questionnaires at baseline, 6 and 12 months.
2) Voice-related quality of life measured using the validated VHI-10 [12]. Participants will self-administer the questionnaires at baseline, 6 and 12 months.
3) Swallowing-related quality of life measured using the validated Eating Assessment Tool (EAT-10 [17]). Participants will self-administer the questionnaires at baseline, 6 and 12 months.
4) Vocal fold vibration measured detected using laryngeal videostroboscopy and graded using the Stroboscopy Research Instrument (SRI) scale [18]. Data will be collected at baseline, 6 and 23 months, anonymised and analysed by three independent trained raters.
5) Perceptual voice quality measured using the GRBAS [11] four point grading scale (0-normal or absence of deviance; 1-slight deviance; 2-moderate deviance and 3-severe deviance) to evaluate 'G'; the Grade of the overall voice quality, 'R'; Roughness or Harshness, 'B'; Breathiness, 'A'; Asthenia and 'S'; Strain. Voice recordings will be collected at baseline, 6 months and 12 months, anonymised and graded by three independent experienced speech therapists trained to enhance the intra- and inter-rater agreement [19].
6) Acoustic voice quality of the participants' voice recording will be captured and measured using the 'On person Rapid Voice Examiner' (OperaVOX™)[20], a specially designed portable voice analysis application that will be loaded onto Apple iPod Touch devices at each recruitment site. The noise-to-harmonic ratio (NHR), harmonic-to-noise (HNR), Jitter, Shimmer, ShimmerdB, Fundamental frequency (Fo), Pitch range and Maximum Phonation Time (MPT) data will be collected at baseline, 6 months and 12 months.
7) Swallow volume, capacity and speed measured using the validated 100ml water Swallow Test [18] at baseline, 6 months and 12 months.
8) Laryngeal muscle activity detected using laryngeal electromyography (LEMG) and graded using the Koufman grading scale [13]. Data obtained will be collected at baseline and 12 months, anonymised and analysed by three independent neurophysiologists.
9) Health economic cost analysis of laryngeal reinnervation and thyroplasty procedures including the costs of the index procedure (staff costs, theatre costs, costs of consumables, recovery costs).
10) Feasibility assessment of conducting a full economic evaluation of laryngeal reinnervation versus thyroplasty alongside the future large scale RCT. Data will be collected to identify: a) the main NHS/Personal Social Services cost components; b) the main costs borne by patients and families;

For peer review only - http://bmjopen.bmj.com/site/about/guidelines.xhtml

c) the resource use and unit cost data required for each of these cost components and how best to source these.

The qualitative secondary outcomes are:

1) Whether members of the Study Management Group (SMG) and recruiters are in equipoise with the treatment arms.
2) Patient views and beliefs on randomisation, recruitment and retention processes.
3) Patient and surgeon/speech therapist views on chosen outcome measures.
4) Recruiter- patient interactions.

Sample size

The sample size of 30 participants (15 per arm) will allow us to demonstrate the feasibility of conducting a full-scale randomised trial in the future. This study is not formally powered to detect differences (although we note that the sample would be able to detect very large effects, e.g. a standardised effect of 1 with approximately 80% power). Rather, the sample size was based on Julious[20] who suggests a group size of at least 12 in order to obtain reasonably precise estimates of an effect and its variance.

Participant flow

Potential participants will attend an initial Ear, Nose and Throat (ENT) consultation with the local Principal Investigator (PI). If they are deemed a potential candidate for the study, the PI will ask the patient for verbal consent to audio-record subsequent conversations where information about the VOCALIST study is given. The PI will then provide the participant information sheet (PIS). After the consultation, the local Research Nurse (RN) will go through the PIS with the patient and answer any questions the patient may have. This conversation will also be audio-recorded and during this conversation the RN will ask the patient to complete written informed pre-consent for the conversations to be audio–recorded and analysed by the qualitative research team.
If the patient is willing to participate in the study, the RN will arrange to call the patient within 1-3 days to book a date for the screening appointment. During the telephone call (also audio-recorded) the RN will answer any further patient questions and give the patient the opportunity to change their mind about study participation if they wish. If the patient confirms they would like to participate, the RN will book the patient's screening appointment at their local site.
At the screening and baseline appointments, informed consent will be obtained by the RN prior to the assessments being performed. Throughout the recruitment process the RN will remind patients that they are under no obligation to enter the study and that they can withdraw at any time, without having to give a reason. No clinical trial procedures will be conducted prior to taking consent from the participant. Consent will not denote enrolment into trial. Figure 1 shows the flow of patients throughout the study and the assessments performed at each visit.

For peer review only - http://bmjopen.bmj.com/site/about/guidelines.xhtml

Randomisation

Participants will be allocated to either of the two operations, see Figure 1, using block randomisation stratified by centre to ensure that approximately equal numbers of patients are allocated to each treatment arm within each centre. The randomisation will be carried out using the web-based service from the company 'Sealed Envelope'. Varying block sizes will be used and these will only be known to the statistician and Sealed Envelope.

Data analysis plan

Quantitative data analysis

Descriptive analyses (means, standard deviations and proportions) will be used to summarise the characteristics of the patients recruited to the study. A consort flow diagram will be produced to show the progress of the participants through the phases of the feasibility study and describe the follow-up at the various time-points. The study dataset will be analysed on an intention-to-treat basis. The primary and secondary outcomes will be analysed descriptively (using either proportions, or means, as appropriate) for each group. Differences between groups will be estimated and presented with 95% confidence intervals. No formal comparisons will be made as the study is not powered for this. Stata 14 will be used for analysis.

Qualitative data analysis

Patient eligibility and recruitment pathway details will be mapped for each centre, to include: the point at which patients receive information about the trial and encounter members of the clinical team, and the timing and frequency of appointments. Logs of eligible and recruited patients will be assembled using simple flow charts and counts to display numbers and percentages of patients at each stage of the eligibility and recruitment process. Audio recordings of the recruitment conversations will be analysed using established techniques of applied conversation analysis [21] to examine how patient consultations proceed, and to identify any instances of interactional trouble which may impact on recruitment.

In depth interviews will be conducted by the Qualitative Researcher with Trial staff (n=10 approximately), including members of the SMG, the CI and those most involved in the design and management of the trial, and with clinical and recruitment staff across the three centres) and participants eligible for recruitment to the study (n=10 approximately, including those who accept and decline randomisation). Interviews will be analysed thematically, using a constant comparison methodology until a point where theoretical saturation is accomplished [22].

Data management

Quantitative data will be collected at the trial sites using paper CRFs and entered centrally by the TM onto the online study database (provided by Sealed Envelope™). Any local data collection or data entry queries raised by local sites will be brought to the attention of the central London team (TM).

For peer review only - http://bmjopen.bmj.com/site/about/guidelines.xhtml

Identification logs, screening logs and enrolment logs will be kept at the trial site in a locked cabinet within a secured room.

Qualitative data will be recorded on encrypted audio recorders and transferred to the University of Bristol's secure Research Data Storage Facility (RDSF). Interviews will be transcribed verbatim, with personal identifiers removed. Files will be stored on the University of Bristol's secure RDSF.

At the end of the study all study-related documents will be archived. The Chief Investigator will archive the study master file for 15 years in line with all relevant legal and statutory requirements. The Principal Investigator at each participating site will archive his/her respective site's study documents for 15 years in line with all relevant legal and statutory requirements.

Study monitoring

The study will be managed by the Study Management Group (SMG) consisting of the co-investigators, site PIs and Patient Representative. They will meet every two months during the set up and duration of the study.

The study will be overseen by the Study Steering Committee (SSC), consisting of an Independent Chair, an independent Clinician, an independent Speech and Language Therapist and a Patient Representative. They will meet bi-annually with the CI and relevant members of the SMG.

Patient and Public Involvement

This research has been developed in response to patient demand for a trial which will deliver the answers they need to make an informed choice regarding their care. Patients and the public have been involved in confirming the need for the research, developing the trial design, choice of outcome measure and wording of patient documentation. Two patient representatives are active members of the SMG and SSC and have been provided with lay research training and support to ensure they understand their role in the research project, remain engaged as active partners and feel confident in their relationships with the research team. These patient representatives will continue to be involved throughout the feasibility study, including assisting with optimising recruitment and retention, interpreting the findings and disseminating the results to their formal and informal patient networks.

Ethics and dissemination

A favourable ethical approval was granted by the NRES - Committee Bromley (reference number 11/LO/0583) prior to commencement of the study. Local approvals for each site have been sought prior to commencement of recruitment. All members of the research team and at each site will be trained in the aspects of Good Clinical Practice (GCP) appropriate to their role in the study.

For peer review only - http://bmjopen.bmj.com/site/about/guidelines.xhtml

To ensure the results from this feasibility study can be taken forward into a plan for a future large scale RCT, there is a need to engage all those involved in voice health including ENT surgeons, speech therapists, voice and singing teachers, GPs and practice nurses. Therefore, results will be disseminated through the publication of articles in peer reviewed medical journals targeted at health professionals involved in the care of patients with voice disorders and presented at national and international scientific meetings. Our findings, and future trial plans, will be discussed with relevant professional organisations including the British Laryngological Association (BLA) and Royal Colleges to ensure they are disseminated to their members through electronic newsletters, websites and national meetings. Our PPI representatives will assist in disseminating results in a suitable lay language to relevant journals and patient information sections of health-related websites, ensuring that patients and the publics are both informed and engaged for the future RCT.

Study status

At the time of manuscript submission, the feasibility study is open to recruitment.

CONCLUSION

Reports on short- and long-term voice outcomes of patients who undergo thyroplasty are sparse and methodologically poor. The quality of the current literature regarding laryngeal reinnervation is also poor. For these reasons, the experienced multi-disciplinary VOCALIST research team have designed this feasibility study to provide reliable information on how to conduct a future multi-centre trial to determine the clinical and cost-effectiveness of laryngeal reinnervation versus thyroplasty for patients with UVFP. The combination of quantitative and qualitative approaches and the support of the BLA and NIHR Clinical Research Network will ensure that all aspects of feasibility are assessed, including patient recruitment strategies and appropriate outcome measures. The lessons learnt in this feasibility study will ensure the success of a future RCT (including whether training courses need to be developed to enable greater numbers of UK surgeons to perform laryngeal reinnervation surgery) ultimately enabling an evidence-based pathway of care to be developed for patients with UVFP.

For peer review only - http://bmjopen.bmj.com/site/about/guidelines.xhtml

DECLARATIONS

Acknowledgements

The authors would like to thank Eilis Longley-Brown and Jaqueline Sayers for their dedicated work as Patient Representatives on the VOCALIST SMG and SSC respectively. The authors would also like to thank the Chair, Mr Julian McGlashan, and members of the SSC as well as the site study staff and VOCALIST participants.

Authors' contributions

MB is the Chief Investigator and led the study design. HB coordinated the grant acquisition, study design, protocol development and drafted the manuscript. PC, MJ, GA, MMB, SM and AS contributed to study design, grant acquisition and protocol development. RY and AD designed and conducted the Patient and Public Involvement work. YK, KH, SH, HK and CW contributed to protocol development. All authors have revised the manuscript and approved the final version.

Funding statement

This paper presents independent research funded by the National Institute for Health Research (NIHR) under its Research for Patient Benefit (RfPB) Programme (Grant Reference Number **PB-PG-1013-32058**). The views expressed are those of the authors and not necessarily those of the NHS, the NIHR or the Department of Health.

This manuscript relates to version 4 of the study protocol, dated 03 March 2017.

Competing interests

The authors declare they have no competing interests.

For peer review only

REFERENCES

1. Misono S, Merati AL. Evidence-based practice: evaluation and management of unilateral vocal fold paralysis. *Otolaryngologic clinics of North America* 2012;45(5):1083-108. doi: 10.1016/j.otc.2012.06.011
2. Fang TJ, Li HY, Gliklich RE, et al. Quality of life measures and predictors for adults with unilateral vocal cord paralysis. *The Laryngoscope* 2008;118(10):1837-41. doi: 10.1097/MLG.0b013e31817e7431
3. Smith ME, Ramig LO, Dromey C, et al. Intensive voice treatment in Parkinson disease: laryngostroboscopic findings. *Journal of voice : official journal of the Voice Foundation* 1995;9(4):453-9.
4. Anderson TD, Spiegel JR, Sataloff RT. Thyroplasty revisions: frequency and predictive factors. *Journal of voice : official journal of the Voice Foundation* 2003;17(3):442-8.
5. Maronian N, Waugh P, Robinson L, et al. Electromyographic findings in recurrent laryngeal nerve reinnervation. *The Annals of otology, rhinology, and laryngology* 2003;112(4):314-23.
6. Uloza V, Pribuisiene R, Saferis V. Multidimensional assessment of functional outcomes of medialization thyroplasty. *European archives of oto-rhino-laryngology : official journal of the European Federation of Oto-Rhino-Laryngological Societies* 2005;262(8):616-21. doi: 10.1007/s00405-004-0755-7
7. Lundy DS, Casiano RR, Xue JW, et al. Thyroplasty type I: short- versus long-term results. *Otolaryngology--head and neck surgery : official journal of American Academy of Otolaryngology-Head and Neck Surgery* 2000;122(4):533-6.
8. Wang W, Chen D, Chen S, et al. Laryngeal reinnervation using ansa cervicalis for thyroid surgery-related unilateral vocal fold paralysis: a long-term outcome analysis of 237 cases. *PloS one* 2011;6(4):e19128. doi: 10.1371/journal.pone.0019128
9. Aynehchi BB, McCoul ED, Sundaram K. Systematic review of laryngeal reinnervation techniques. *Otolaryngology--head and neck surgery : official journal of American Academy of Otolaryngology-Head and Neck Surgery* 2010;143(6):749-59. doi: 10.1016/j.otohns.2010.09.031
10. Donovan JL, Rooshenas L, Jepson M, et al. Optimising recruitment and informed consent in randomised controlled trials: the development and implementation of the Quintet Recruitment Intervention (QRI). *Trials* 2016;17(1):283. doi: 10.1186/s13063-016-1391-4
11. Hirano M. Clinical Examination of Voice. Wien: Springer-Verlag 1981:100.
12. Rosen CA, Lee AS, Osborne J, et al. Development and validation of the voice handicap index-10. *The Laryngoscope* 2004;114(9):1549-56. doi: 10.1097/00005537-200409000-00009
13. Koufman KA. Laryngeal electromyography in clinical practice: indications, techniques, and interpretation. *Phonoscope* 1998;1:57-70.
14. Crumley RL. Teflon versus thyroplasty versus nerve transfer: a comparison. *The Annals of otology, rhinology, and laryngology* 1990;99(10 Pt 1):759-63.
15. Montgomery WW, Blaugrund SM, Varvares MA. Thyroplasty: a new approach. *The Annals of otology, rhinology, and laryngology* 1993;102(8 Pt 1):571-9.
16. EQ-5D http://www.euroqol.org/ [
17. Belafsky PC, Mouadeb DA, Rees CJ, et al. Validity and reliability of the Eating Assessment Tool (EAT-10). *The Annals of otology, rhinology, and laryngology* 2008;117(12):919-24.
18. Wu MC, Chang YC, Wang TG, et al. Evaluating swallowing dysfunction using a 100-ml water swallowing test. *Dysphagia* 2004;19(1):43-7. doi: 10.1007/s00455-003-0030-x
19. Dejonckere PH, Bradley P, Clemente P, et al. A basic protocol for functional assessment of voice pathology, especially for investigating the efficacy of (phonosurgical) treatments and evaluating new assessment techniques. Guideline elaborated by the Committee on Phoniatrics of the European Laryngological Society (ELS). *European archives of oto-rhino-*

For peer review only

laryngology : official journal of the European Federation of Oto-Rhino-Laryngological Societies 2001;258(2):77-82.
20. Mat Baki M, Wood G, Alston M, et al. Reliability of OperaVOX against Multidimensional Voice Program (MDVP). *Clin Otolaryngol* 2015;40(1):22-8. doi: 10.1111/coa.12313
21. Sidnell J, Stivers T. The Handbook of Conversation Analysis: Wiley-Blackwell 2013.
22. Goetz JP. Ethnographic Research and the Problem of Data Reduction. *Anthr Educ Q* 1981;12(1):51-70.

For peer review only

For peer review only - http://bmjopen.bmj.com/site/about/guidelines.xhtml

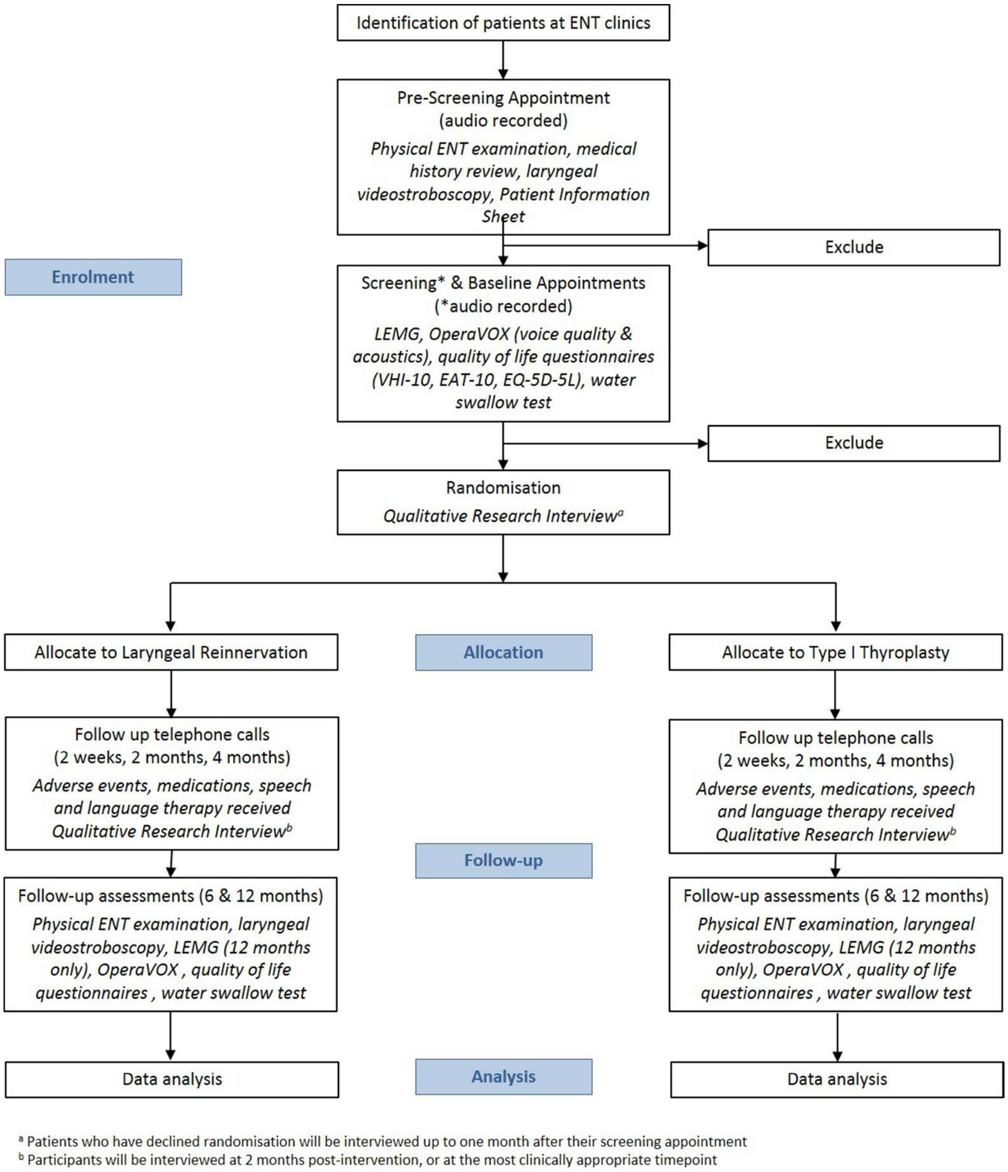

Figure 1. VOCALIST feasibilty study participant flow diagram

204x240mm (150 x 150 DPI)

SPIRIT

STANDARD PROTOCOL ITEMS: RECOMMENDATIONS FOR INTERVENTIONAL TRIALS

SPIRIT 2013 Checklist: Recommended items to address in a clinical trial protocol and related documents*

Section/item	Item No	Description	Addressed on page number
**Administrative information**			
Title	1	Descriptive title identifying the study design, population, interventions, and, if applicable, trial acronym	1
Trial registration	2a	Trial identifier and registry name. If not yet registered, name of intended registry	1
	2b	All items from the World Health Organization Trial Registration Data Set	1
Protocol version	3	Date and version identifier	1
Funding	4	Sources and types of financial, material, and other support	1 & 39
Roles and responsibilities	5a	Names, affiliations, and roles of protocol contributors	9
	5b	Name and contact information for the trial sponsor	9
	5c	Role of study sponsor and funders, if any, in study design; collection, management, analysis, and interpretation of data; writing of the report; and the decision to submit the report for publication, including whether they will have ultimate authority over any of these activities	40
	5d	Composition, roles, and responsibilities of the coordinating centre, steering committee, endpoint adjudication committee, data management team, and other individuals or groups overseeing the trial, if applicable (see Item 21a for data monitoring committee)	38

For peer review only - http://bmjopen.bmj.com/site/about/guidelines.xhtml

For peer review only

**Introduction**			
Background and rationale	6a	Description of research question and justification for undertaking the trial, including summary of relevant studies (published and unpublished) examining benefits and harms for each intervention	13-14
	6b	Explanation for choice of comparators	13-14
Objectives	7	Specific objectives or hypotheses	14
Trial design	8	Description of trial design including type of trial (eg, parallel group, crossover, factorial, single group), allocation ratio, and framework (eg, superiority, equivalence, noninferiority, exploratory)	15
**Methods: Participants, interventions, and outcomes**			
Study setting	9	Description of study settings (eg, community clinic, academic hospital) and list of countries where data will be collected. Reference to where list of study sites can be obtained	16
Eligibility criteria	10	Inclusion and exclusion criteria for participants. If applicable, eligibility criteria for study centres and individuals who will perform the interventions (eg, surgeons, psychotherapists)	15-16
Interventions	11a	Interventions for each group with sufficient detail to allow replication, including how and when they will be administered	19, 22-23 & 42-45
	11b	Criteria for discontinuing or modifying allocated interventions for a given trial participant (eg, drug dose change in response to harms, participant request, or improving/worsening disease)	22
	11c	Strategies to improve adherence to intervention protocols, and any procedures for monitoring adherence (eg, drug tablet return, laboratory tests)	39
	11d	Relevant concomitant care and interventions that are permitted or prohibited during the trial	23
Outcomes	12	Primary, secondary, and other outcomes, including the specific measurement variable (eg, systolic blood pressure), analysis metric (eg, change from baseline, final value, time to event), method of aggregation (eg, median, proportion), and time point for each outcome. Explanation of the clinical relevance of chosen efficacy and harm outcomes is strongly recommended	34-35
Participant timeline	13	Time schedule of enrolment, interventions (including any run-ins and washouts), assessments, and visits for participants. A schematic diagram is highly recommended (see Figure)	21

For peer review only - http://bmjopen.bmj.com/site/about/guidelines.xhtml

Sample size	14	Estimated number of participants needed to achieve study objectives and how it was determined, including clinical and statistical assumptions supporting any sample size calculations	35-36
Recruitment	15	Strategies for achieving adequate participant enrolment to reach target sample size	36
**Methods: Assignment of interventions (for controlled trials)**			
Allocation:			
Sequence generation	16a	Method of generating the allocation sequence (eg, computer-generated random numbers), and list of any factors for stratification. To reduce predictability of a random sequence, details of any planned restriction (eg, blocking) should be provided in a separate document that is unavailable to those who enrol participants or assign interventions	38
Allocation concealment mechanism	16b	Mechanism of implementing the allocation sequence (eg, central telephone; sequentially numbered, opaque, sealed envelopes), describing any steps to conceal the sequence until interventions are assigned	38
Implementation	16c	Who will generate the allocation sequence, who will enrol participants, and who will assign participants to interventions	38
Blinding (masking)	17a	Who will be blinded after assignment to interventions (eg, trial participants, care providers, outcome assessors, data analysts), and how	15
	17b	If blinded, circumstances under which unblinding is permissible, and procedure for revealing a participant's allocated intervention during the trial	18
**Methods: Data collection, management, and analysis**			
Data collection methods	18a	Plans for assessment and collection of outcome, baseline, and other trial data, including any related processes to promote data quality (eg, duplicate measurements, training of assessors) and a description of study instruments (eg, questionnaires, laboratory tests) along with their reliability and validity, if known. Reference to where data collection forms can be found, if not in the protocol	30-33
	18b	Plans to promote participant retention and complete follow-up, including list of any outcome data to be collected for participants who discontinue or deviate from intervention protocols	22

For peer review only - http://bmjopen.bmj.com/site/about/guidelines.xhtml

Data management	19	Plans for data entry, coding, security, and storage, including any related processes to promote data quality (eg, double data entry; range checks for data values). Reference to where details of data management procedures can be found, if not in the protocol	33-34
Statistical methods	20a	Statistical methods for analysing primary and secondary outcomes. Reference to where other details of the statistical analysis plan can be found, if not in the protocol	36-38
	20b	Methods for any additional analyses (eg, subgroup and adjusted analyses)	36-38
	20c	Definition of analysis population relating to protocol non-adherence (eg, as randomised analysis), and any statistical methods to handle missing data (eg, multiple imputation)	36-38
**Methods: Monitoring**			
Data monitoring	21a	Composition of data monitoring committee (DMC); summary of its role and reporting structure; statement of whether it is independent from the sponsor and competing interests; and reference to where further details about its charter can be found, if not in the protocol. Alternatively, an explanation of why a DMC is not needed	39
	21b	Description of any interim analyses and stopping guidelines, including who will have access to these interim results and make the final decision to terminate the trial	38
Harms	22	Plans for collecting, assessing, reporting, and managing solicited and spontaneously reported adverse events and other unintended effects of trial interventions or trial conduct	25-29
Auditing	23	Frequency and procedures for auditing trial conduct, if any, and whether the process will be independent from investigators and the sponsor	N/A
**Ethics and dissemination**			
Research ethics approval	24	Plans for seeking research ethics committee/institutional review board (REC/IRB) approval	38-39
Protocol amendments	25	Plans for communicating important protocol modifications (eg, changes to eligibility criteria, outcomes, analyses) to relevant parties (eg, investigators, REC/IRBs, trial participants, trial registries, journals, regulators)	38-39

For peer review only - http://bmjopen.bmj.com/site/about/guidelines.xhtml

Consent or assent	26a	Who will obtain informed consent or assent from potential trial participants or authorised surrogates, and how (see Item 32)	17
	26b	Additional consent provisions for collection and use of participant data and biological specimens in ancillary studies, if applicable	N/A
Confidentiality	27	How personal information about potential and enrolled participants will be collected, shared, and maintained in order to protect confidentiality before, during, and after the trial	30
Declaration of interests	28	Financial and other competing interests for principal investigators for the overall trial and each study site	N/A
Access to data	29	Statement of who will have access to the final trial dataset, and disclosure of contractual agreements that limit such access for investigators	38
Ancillary and post-trial care	30	Provisions, if any, for ancillary and post-trial care, and for compensation to those who suffer harm from trial participation	29
Dissemination policy	31a	Plans for investigators and sponsor to communicate trial results to participants, healthcare professionals, the public, and other relevant groups (eg, via publication, reporting in results databases, or other data sharing arrangements), including any publication restrictions	40
	31b	Authorship eligibility guidelines and any intended use of professional writers	N/A
	31c	Plans, if any, for granting public access to the full protocol, participant-level dataset, and statistical code	N/A
**Appendices**			
Informed consent materials	32	Model consent form and other related documentation given to participants and authorised surrogates	N/A
Biological specimens	33	Plans for collection, laboratory evaluation, and storage of biological specimens for genetic or molecular analysis in the current trial and for future use in ancillary studies, if applicable	N/A

*It is strongly recommended that this checklist be read in conjunction with the SPIRIT 2013 Explanation & Elaboration for important clarification on the items. Amendments to the protocol should be tracked and dated. The SPIRIT checklist is copyrighted by the SPIRIT Group under the Creative Commons "Attribution-NonCommercial-NoDerivs 3.0 Unported" license.

For peer review only - http://bmjopen.bmj.com/site/about/guidelines.xhtml

BMJ Open

Does Laryngeal Reinnervation or Type I Thyroplasty give better voice results for patients with Unilateral Vocal Fold Paralysis (VOCALIST): study protocol for a feasibility randomised controlled trial

Journal:	*BMJ Open*
Manuscript ID	bmjopen-2017-016871.R1
Article Type:	Protocol
Date Submitted by the Author:	20-Jun-2017
Complete List of Authors:	Blackshaw, Helen; University College London Ear Institute, evidENT<br>Carding, Paul; Australian Catholic University Faculty of Health Sciences<br>Jepson, Marcus; University of Bristol School of Social and Community Medicine<br>Mat Baki, Marina; Pusat Perubatan Universiti Kebangsaan Malaysia<br>Ambler, Gareth; University College London<br>Schilder, Anne; University College London Ear Institute, evidENT<br>Morris, Stephen; University College London, Department of Applied Health Research<br>Degun, Aneeka; University College London Ear Institute, evidENT<br>Yu, Rosamund; University College London Hospitals NHS Foundation Trust, Biomedical Research Centre<br>Husbands, Samantha; University of Bristol School of Social and Community Medicine<br>Knowles, Helen; University College London Hospitals NHS Foundation Trust, Royal National Throat, Nose and Ear Hospital<br>Walton, Chloe; Australian Catholic University Faculty of Health Sciences<br>Karagama, Yakubu; Central Manchester University Hospitals NHS Foundation Trust, Manchester Royal Infirmary<br>Heathcote, Kate; Poole Hospital NHS Foundation Trust<br>Birchall, Martin; University College London Ear Institute
**Primary Subject Heading**:	Ear, nose and throat/otolaryngology
Secondary Subject Heading:	Surgery
Keywords:	Adult otolaryngology < OTOLARYNGOLOGY, Laryngology < OTOLARYNGOLOGY, Speech pathology < OTOLARYNGOLOGY, Otolaryngology < SURGERY, Clinical trials < THERAPEUTICS

SCHOLARONE™
Manuscripts

Does Laryngeal Reinnervation or Type I Thyroplasty give better voice results for patients with Unilateral Vocal Fold Paralysis (VOCALIST): study protocol for a feasibility randomised controlled trial

Helen Blackshaw[1], Paul Carding[2], Marcus Jepson[3], Marina Mat Baki[4], Gareth Ambler[5], Anne Schilder[1], Stephen Morris[6], Aneeka Degun[1], Rosamund Yu[7], Samantha Husbands[3], Helen Knowles[8], Chloe Walton[2], Yakubu Karagama[9], Kate Heathcote[10] and Martin Birchall[1]

[1] Ear Institute, University College London, UK

[2] School of Allied Health, Australian National Catholic University, Australia

[3] School of Social and Community Medicine, University of Bristol, UK

[4] Faculty of Medicine, National University of Malaysia, Malaysia

[5] Statistical Science, University College London, UK

[6] Department of Applied Health Research, University of London, UK

[7] UCL Hospitals Biomedical Research Centre, University College London Hospitals NHS Foundation Trust, UK

[8] Royal National Throat, Nose and Ear Hospital, University College London Hospitals NHS Foundation Trust, UK

[9] Department of Otolaryngology-Head & Neck Surgery, Manchester Royal Infirmary, UK

[10] Department of Otolaryngology, Poole Hospital, UK

Corresponding author:

Dr Helen Blackshaw

Ear Institute, University College London, Royal National Throat, Nose and Ear Hospital, 330 Grays Inn Road, London WC1X 8DA

h.blackshaw@ucl.ac.uk

020 3108 9326

Word count (excluding title page, abstract, figures and references): 3771 words

For peer review only - http://bmjopen.bmj.com/site/about/guidelines.xhtml

ABSTRACT

Introduction: A functioning voice is essential for normal human communication. A good voice requires two moving vocal folds; if one fold is paralysed (unilateral vocal fold paralysis-UVFP) people suffer from a breathy, weak voice that tires easily and is unable to function normally. UVFP can also result in choking and breathlessness. Current treatment for adults with UVFP is speech therapy to stimulate recovery of vocal fold (VF) motion or function and/or injection of the paralysed VF with a material to move it into a more favourable position for the functioning VF to close against. When these therapies are unsuccessful, or only provide temporary relief, surgery is offered. Two available surgical techniques are; i) surgical medialisation; placing an implant near the paralysed VF to move it to the middle (thyroplasty) and/or repositioning the cartilage (arytenoid adduction), or ii) restoring the nerve supply to the VF (laryngeal reinnervation). Currently there is limited evidence to determine which surgery should be offered to adults with UVFP.

Methods and analysis: A feasibility study to test the practicality of running a multi-centre, randomised clinical trial of surgery for UVFP, including; a) a qualitative study to understand the recruitment process and how it operates in clinical centres; and b) a small randomised trial of 30 participants recruited at 3 UK sites comparing non-selective laryngeal reinnervation to type I thyroplasty. Participants will be followed up for 12 months. The primary outcome focuses on recruitment and retention, with secondary outcomes covering voice, swallowing and quality of life.

Ethics and dissemination: Ethical approval was received from NRES - Committee Bromley (reference 11/LO/0583). In addition to dissemination of results through presentation and publication of peer-reviewed articles, results will be shared with key clinician and patient groups required to develop the future large scale RCT.

Trial registration: ISRCTN90201732; 16 December 2015.

For peer review only

For peer review only - http://bmjopen.bmj.com/site/about/guidelines.xhtml

STRENGTHS AND LIMITATIONS OF THIS STUDY

- This is the first clinical trial to be run in the UK to compare the effectiveness of two surgical approaches for restoration of laryngeal function in adults with Unilateral Vocal Fold Paralysis (UVFP)
- This study has been developed in response to; a) patient demand and b) a systematic review that reported a low quality of current literature available on laryngeal reinnervation and proposed that a formal prospective trial was required using standardised, internationally accepted outcomes.
- A similar trial, comparing laryngeal reinnervation and type I thyroplasty in adults with UVFP, was previously run in the US, but was suspended prematurely due to irregularity in obtaining inform consent and low accrual. We acknowledge the challenges associated with recruiting to a phase III surgical study of this type and have sought to overcome them by; a) learning from this previous trial; b) designing this rigorous feasibility study to be conducted prior to running a larger scale phase III RCT; and c) involving UVFP patients throughout the feasibility study design, set up, analysis and dissemination processes.
- Our research team is multi-disciplinary and our study design incorporates both qualitative and quantitative components.
- Whilst our study focuses on voice-related criteria for inclusion, our design includes outcomes relating to both the voice and swallowing.
- Although participants and study personnel are not blinded to their allocated treatment group, the speech therapist and neurophysiologist outcome raters are blinded.
- Very few ENT surgeons in the UK are trained to perform laryngeal reinnervation and so our study is limited to recruiting at those relevant UK sites.
- Whilst we aim to follow-up all participants for 12 months, budget and time constraints may limit some of the latterly-recruited participant follow-up to 6 months. However, all patients are followed up clinically in the long-term.

For peer review only

For peer review only - http://bmjopen.bmj.com/site/about/guidelines.xhtml

INTRODUCTION

The larynx provides the main tool for human social interaction. The vocal folds are innervated by the recurrent laryngeal nerve (RLN) and when this nerve supply to one vocal fold is interrupted, unilateral vocal fold paralysis (UVFP) ensues. Patients suffering with UVFP typically experience a hoarse, weak voice [1] that tires easily, with some also suffering from breathing and swallowing problems. Quality of life of these patients is significantly reduced and social and employment opportunities are compromised [2 3] .Therefore, optimum rehabilitation of the paralysed larynx is important for health and wealth alike.

The aim of treatment for UVFP is to achieve optimum vocal fold (VF) closure for voice production. Where speech therapy alone is insufficient to improve the voice, surgical treatment is undertaken. Whilst short-term relief of symptoms can be achieved via injection of the paralysed VF with a material to improve its position for the functioning VF to close against it, permanent results are only possible by two types of operation: surgical medialisation and laryngeal reinnervation. One method of the former is to insert a synthetic implant to move the paralysed VF into a better position for speaking (type 1 thyroplasty), whilst the latter re-supplies nerves to improve muscle bulk and tone (non-selective laryngeal reinnervation). Type I thyroplasty is a one-hour operation under local anaesthetic where the medium grade silastic implant is inserted through a window in the cartilage lamina of the larynx. This implant medialises the paralysed vocal fold in the midline position allowing the normal opposite vocal fold to make firm contact and produce a stronger voice. However, voice improvement may be short-term due to progressive atrophy of the denervated laryngeal muscles [4]. Furthermore, this procedure does not restore vocal fold tension, meaning that pitch variation remains suboptimal [5 6]. Reports on short- and long-term voice outcomes of patients with thyroplasty are sparse and methodologically poor [7]. The newer approach to restoring laryngeal function in UVFP patients, non-selective laryngeal reinnervation, is a two-hour operation under general anaesthesia in which the injured RLN and the donor nerve are identified and anastomosed. Its aim is to restore innervation to the larynx by ‘borrowing’ the activity of other motor nerves, specifically the ansa cervicalis. As such it may re-establish the tone and bulk of the vocal folds during speech and re-enable pitch control resulting in normal or near normal voice. The operation is safe and results in sustained improvement of glottal closure, maintenance of the vocal fold edge and significant improvements in voice quality have been reported [8].

A systematic review of studies on laryngeal reinnervation revealed that the quality of current literature is low and a formal prospective trial was recommended using standardised, internationally accepted outcomes [9]. Before embarking on a trial that will use public resources and the time of patients and healthcare staff, we need to confirm that running a trial is feasible and that the chosen outcome measures truly reflect voice quality and patients’ perceptions [10]. For these reasons, and others given above, a feasibility study is critical prior to designing, with patients, a multi-centre, phase III randomised clinical trial of treatment for this disabling condition.

For peer review only - http://bmjopen.bmj.com/site/about/guidelines.xhtml

METHODS AND ANALYSIS

Objectives

The primary research objective is to determine the feasibility of performing a multi-centre phase III randomised controlled trial of laryngeal reinnervation versus type I thyroplasty for adult patients with UVFP.

The secondary research objectives are to test the feasibility of:

1) The multicentre recruitment process, including continuous improvement to the process based on qualitative methodology.
2) Recruiters being able to present true equipoise with the treatment arms.
3) The randomisation processes and to investigate reasons for any difficulties that affect recruitment.
4) The utility of the following characteristics of the proposed primary and secondary outcomes: variability across patients, variability over time, differences in outcome between randomised groups over time.
5) Process of follow up visits.
6) Means of gathering health economics and health-related quality of life data suitable for measuring cost-effectiveness.

Study design

The feasibility study will consist of two research approaches run in parallel;

1) Qualitative: The QuinteT Recruitment Intervention (QRI) to understand the recruitment process and how it operates in clinical centres, so that sources of recruitment difficulties can be identified and suggestions made to change aspects of design, conduct, organisation or training that could then lead to improvements in recruitment [11].

2) Quantitative: A small randomised surgical trial of 30 patients to compare non-selective laryngeal reinnervation to type I thyroplasty. Patients will be randomised in a 1:1 ratio.

The study is single blinded whereby the raters of the secondary quantitative outcome of acoustic measures will be blinded to the timepoint of the data recordings (preoperative or postoperative) and blinded to surgical intervention (laryngeal reinnervation or type I thyroplasty).

Participants

Inclusion criteria

1) UVFP due to unilateral recurrent laryngeal nerve paralysis of traumatic, iatrogenic or idiopathic origin of between 6 and 60 months duration. Or symptoms that have not sufficiently improved with speech therapy alone, as determined by the patient and agreed by a multidisciplinary clinical team, after 6 months and pending a surgical decision.

For peer review only - http://bmjopen.bmj.com/site/about/guidelines.xhtml

2) Age from 18 to 70 years old.
3) Male or female.
4) Able to provide informed consent.
5) A significant voice disorder as measured by perceptual rating (GRBAS Grade ≥2)[12] and Voice Handicap Index (VHI-10 score >16) [13].
6) Common laryngeal electromyography (LEMG, neurophysiological) criteria (Koufman Grades 2-5)[14] in either the thyroarytenoid (TA) or posterior cricoarytenoid (PCA) muscle on the paralysed side.

Exclusion criteria

1) Impaired vocal fold mobility but a normal EMG (Koufman Grade I).
2) Severe lung disorders.
3) Structural vocal fold lesions such as polyp.
4) Previous laryngeal framework surgery.
5) Cricoarytenoid joint fixation (CAJF).
6) Significant non-laryngeal speech abnormality (severe dysarthria determined by a panel of trained speech therapists).
7) Previous Level 2, 3 or 4 thyroid neck dissection.
8) Previous ipsilateral surgical neck dissection.
9) Previous radiotherapy to the head and neck.
10) Laryngeal injection of a rapidly absorbable material in the last 6 months.
11) Previous laryngeal injection of a non-rapidly absorbable material (e.g. bioplastics, VOX).
12) Neuromuscular disease affecting the larynx or multiple cranial nerve palsies.

Setting

The study is sponsored by University College London Hospitals NHS Foundation Trust. Potential participants will be recruited from three UK hospitals; the Royal National Throat, Nose and Ear Hospital (University College London Hospitals NHS Foundation Trust), Manchester Royal Infirmary (Central Manchester University Hospitals NHS Foundation Trust) and Poole Hospital (Poole Hospital NHS Foundation Trust). The study is run through the University College London evidENT team, with qualitative data analysed by the University of Bristol and quantitative data analysed by University College London and Australian Catholic University.

Interventions

Laryngeal Reinnervation: The surgical technique is an unselective reinnervation [15] that aims to improve the muscle tone and bulk. Performed under general anaesthesia, the injured RLN and the donor nerve are identified and anastomosed.
Type 1 Thyroplasty: This medialisation/augmentation technique is a static technique [16], performed under local anaesthesia that aims to improve the positioning of the paralysed vocal fold. It uses a medium grade silastic implant, cut by the surgeon into the correct size for the patient. This size is determined intraoperatively by using a measuring device while listening and visualising the larynx

For peer review only - http://bmjopen.bmj.com/site/about/guidelines.xhtml

For peer review only

with flexible fibreoptic scope simultaneously. Concurrent arytenoid adduction is permitted to be performed in cases where there is a significant posterior gap that cannot be approximated with the silicon block alone.

Outcome measures

The primary outcomes are, from those patients who were eligible to enter the study:

1) Whether the patient was randomised (yes/no).
2) Whether the participant successfully received the allocated operation (yes/no).
3) Whether the participant completed the trial (yes/no).

The quantitative secondary outcomes are:

1) General health status measured using the validated EQ-5D-5L [17] questionnaire. Participants will self-administer the questionnaires at baseline, 6 and 12 months.
2) Voice-related quality of life measured using the validated VHI-10 [13]. Participants will self-administer the questionnaires at baseline, 6 and 12 months.
3) Swallowing-related quality of life measured using the validated Eating Assessment Tool (EAT-10 [18]). Participants will self-administer the questionnaires at baseline, 6 and 12 months.
4) Vocal fold vibration measured detected using laryngeal videostroboscopy and graded using the Stroboscopy Research Instrument (SRI) scale [19]. Data will be collected at baseline, 6 and 12 months, anonymised and analysed by three independent trained raters.
5) Perceptual voice quality measured using the GRBAS [12] four point grading scale (0-normal or absence of deviance; 1-slight deviance; 2-moderate deviance and 3-severe deviance) to evaluate 'G'; the Grade of the overall voice quality, 'R'; Roughness or Harshness, 'B'; Breathiness, 'A'; Asthenia and 'S'; Strain. Voice recordings will be collected at baseline, 6 months and 12 months, anonymised and graded by three independent experienced speech therapists trained to enhance the intra- and inter-rater agreement [20].
6) Acoustic voice quality of the participants' voice recording will be captured and measured using the 'On person Rapid Voice Examiner' (OperaVOX™)[21], a specially designed portable voice analysis application that will be loaded onto Apple iPod Touch devices at each recruitment site. The noise-to-harmonic ratio (NHR), harmonic-to-noise (HNR), Jitter, Shimmer, ShimmerdB, Fundamental frequency (Fo), Pitch range and Maximum Phonation Time (MPT) data will be collected at baseline, 6 months and 12 months.
7) Swallow volume, capacity and speed measured using the validated 100ml water Swallow Test [19] at baseline, 6 months and 12 months.
8) Laryngeal muscle activity detected using laryngeal electromyography (LEMG) and graded using the Koufman grading scale [14]. Data obtained will be collected at baseline and 12 months, anonymised and analysed by three independent neurophysiologists.
9) Health economic cost analysis of laryngeal reinnervation and thyroplasty procedures including the costs of the index procedure (staff costs, theatre costs, costs of consumables, recovery costs).
10) Feasibility assessment of conducting a full economic evaluation of laryngeal reinnervation versus thyroplasty alongside the future large scale RCT. Data will be collected to identify: a) the main

For peer review only - http://bmjopen.bmj.com/site/about/guidelines.xhtml

NHS/Personal Social Services cost components; b) the main costs borne by patients and families; c) the resource use and unit cost data required for each of these cost components and how best to source these.

The qualitative secondary outcomes are:

1) Whether members of the Study Management Group (SMG) and recruiters are in equipoise with the treatment arms.
2) Patient views and beliefs on randomisation, recruitment and retention processes.
3) Patient and surgeon/speech therapist views on chosen outcome measures.
4) Recruiter- patient interactions.

Sample size

The sample size of 30 participants (15 per arm) will allow us to demonstrate the feasibility of conducting a full-scale randomised trial in the future. This study is not formally powered to detect differences (although we note that the sample would be able to detect very large effects, e.g. a standardised effect of 1 with approximately 80% power). Rather, the sample size was based on Julious[20] who suggests a group size of at least 12 in order to obtain reasonably precise estimates of an effect and its variance.

Participant flow

During the 15 month recruitment period, potential participants will attend an initial Ear, Nose and Throat (ENT) consultation with the local Principal Investigator (PI). If they are deemed a potential candidate for the study, the PI will ask the patient for verbal consent to audio-record subsequent conversations where information about the VOCALIST study is given. The PI will then provide the participant information sheet (PIS). After the consultation, the local Research Nurse (RN) will go through the PIS with the patient and answer any questions the patient may have. This conversation will also be audio-recorded and during this conversation the RN will ask the patient to complete written informed pre-consent for the conversations to be audio–recorded and analysed by the qualitative research team.

If the patient is willing to participate in the study, the RN will arrange to call the patient within 1-3 days to book a date for the screening appointment. During the telephone call (also audio-recorded) the RN will answer any further patient questions and give the patient the opportunity to change their mind about study participation if they wish. If the patient confirms they would like to participate, the RN will book the patient’s screening appointment at their local site.

At the screening and baseline appointments, informed consent will be obtained by the RN prior to the assessments being performed. Throughout the recruitment process the RN will remind patients that they are under no obligation to enter the study and that they can withdraw at any time, without having to give a reason. No clinical trial procedures will be conducted prior to taking consent from the participant. Consent will not denote enrolment into trial. Figure 1 shows the flow of patients through the study and the assessments performed at each visit.

For peer review only - http://bmjopen.bmj.com/site/about/guidelines.xhtml

For peer review only

Randomisation

Participants will be allocated to either of the two operations, see Figure 1, using block randomisation stratified by centre to ensure that approximately equal numbers of patients are allocated to each treatment arm within each centre. The randomisation will be carried out using the web-based service from the company 'Sealed Envelope'. Varying block sizes will be used and these will only be known to the statistician and Sealed Envelope.

Data analysis plan

Quantitative data analysis

Descriptive analyses (means, standard deviations and proportions) will be used to summarise the characteristics of the patients recruited to the study. A consort flow diagram will be produced to show the progress of the participants through the phases of the feasibility study and describe the follow-up at the various time-points. The study dataset will be analysed on an intention-to-treat basis. The primary and secondary outcomes will be analysed descriptively (using either proportions, or means, as appropriate) for each group. Differences between groups will be estimated and presented with 95% confidence intervals. No formal comparisons will be made as the study is not powered for this. Stata 14 will be used for analysis.

Qualitative data analysis

Patient eligibility and recruitment pathway details will be mapped for each centre, to include: the point at which patients receive information about the trial and encounter members of the clinical team, and the timing and frequency of appointments. Logs of eligible and recruited patients will be assembled using simple flow charts and counts to display numbers and percentages of patients at each stage of the eligibility and recruitment process. Audio recordings of the recruitment conversations will be analysed using established techniques of applied conversation analysis [22] to examine how patient consultations proceed, and to identify any instances of interactional trouble which may impact on recruitment.

In depth interviews will be conducted by the Qualitative Researcher with Trial staff (n=10 approximately), including members of the SMG, the CI and those most involved in the design and management of the trial, and with clinical and recruitment staff across the three centres) and participants eligible for recruitment to the study (n=10 approximately, including those who accept and decline randomisation). Interviews will be analysed thematically, using a constant comparison methodology until a point where theoretical saturation is accomplished [23].

Data management

Quantitative data will be collected at the trial sites using paper CRFs and entered centrally by the TM onto the online study database (provided by Sealed Envelope™). Any local data collection or data entry queries raised by local sites will be brought to the attention of the central London team (TM).

For peer review only - http://bmjopen.bmj.com/site/about/guidelines.xhtml

Identification logs, screening logs and enrolment logs will be kept at the trial site in a locked cabinet within a secured room.

Qualitative data will be recorded on encrypted audio recorders and transferred to the University of Bristol's secure Research Data Storage Facility (RDSF). Interviews will be transcribed verbatim, with personal identifiers removed. Files will be stored on the University of Bristol's secure RDSF.

At the end of the study all study-related documents will be archived. The Chief Investigator will archive the study master file for 15 years in line with all relevant legal and statutory requirements. The Principal Investigator at each participating site will archive his/her respective site's study documents for 15 years in line with all relevant legal and statutory requirements.

Study monitoring

The study will be managed by the Study Management Group (SMG) consisting of the co-investigators, site PIs and Patient Representative. They will meet every two months during the set up and duration of the study.

The study will be overseen by the Study Steering Committee (SSC), consisting of an Independent Chair, an independent Clinician, an independent Speech and Language Therapist and a Patient Representative. They will meet bi-annually with the CI and relevant members of the SMG.

Patient and Public Involvement

This research has been developed in response to patient demand for a trial which will deliver the answers they need to make an informed choice regarding their care. Patients and the public have been involved in confirming the need for the research, developing the trial design, choice of outcome measure and wording of patient documentation. Two patient representatives are active members of the SMG and SSC and have been provided with lay research training and support to ensure they understand their role in the research project, remain engaged as active partners and feel confident in their relationships with the research team. These patient representatives will continue to be involved throughout the feasibility study, including assisting with optimising recruitment and retention, interpreting the findings and disseminating the results to their formal and informal patient networks.

Ethics and dissemination

A favourable ethical approval was granted by the NRES - Committee Bromley (reference number 11/LO/0583) prior to commencement of the study. Local approvals for each site have been sought prior to commencement of recruitment. All members of the research team and at each site will be trained in the aspects of Good Clinical Practice (GCP) appropriate to their role in the study.

For peer review only - http://bmjopen.bmj.com/site/about/guidelines.xhtml

For peer review only

To ensure the results from this feasibility study can be taken forward into a plan for a future large scale RCT, there is a need to engage all those involved in voice health including ENT surgeons, speech therapists, voice and singing teachers, GPs and practice nurses. Therefore, results will be disseminated through the publication of articles in peer reviewed medical journals targeted at health professionals involved in the care of patients with voice disorders and presented at national and international scientific meetings. Our findings, and future trial plans, will be discussed with relevant professional organisations including the British Laryngological Association (BLA) and Royal Colleges to ensure they are disseminated to their members through electronic newsletters, websites and national meetings. Our PPI representatives will assist in disseminating results in a suitable lay language to relevant journals and patient information sections of health-related websites, ensuring that patients and the publics are both informed and engaged for the future RCT.

Study status

At the time of manuscript submission, the feasibility study is open to recruitment.

CONCLUSION

Reports on short- and long-term voice outcomes of patients who undergo thyroplasty are sparse and methodologically poor. The quality of the current literature regarding laryngeal reinnervation is also poor. For these reasons, the experienced multi-disciplinary VOCALIST research team have designed this feasibility study to provide reliable information on how to conduct a future multi-centre trial to determine the clinical and cost-effectiveness of laryngeal reinnervation versus thyroplasty for patients with UVFP. The combination of quantitative and qualitative approaches and the support of the BLA and NIHR Clinical Research Network will ensure that all aspects of feasibility are assessed, including patient recruitment strategies and appropriate outcome measures. The lessons learnt in this feasibility study will ensure the success of a future RCT (including whether training courses need to be developed to enable greater numbers of UK surgeons to perform laryngeal reinnervation surgery) ultimately enabling an evidence-based pathway of care to be developed for patients with UVFP.

For peer review only - http://bmjopen.bmj.com/site/about/guidelines.xhtml

DECLARATIONS

Acknowledgements

The authors would like to thank Eilis Longley-Brown and Jaqueline Sayers for their dedicated work as Patient Representatives on the VOCALIST SMG and SSC respectively. The authors would also like to thank the Chair, Mr Julian McGlashan, and members of the SSC as well as the site study staff and VOCALIST participants.

Authors' contributions

MB is the Chief Investigator and led the study design. HB coordinated the grant acquisition, study design, protocol development and drafted the manuscript. PC, MJ, GA, MMB, SM and AS contributed to study design, grant acquisition and protocol development. RY and AD designed and conducted the Patient and Public Involvement work. YK, KH, SH, HK and CW contributed to protocol development. All authors have revised the manuscript and approved the final version.

Funding statement

This paper presents independent research funded by the National Institute for Health Research (NIHR) under its Research for Patient Benefit (RfPB) Programme (Grant Reference Number **PB-PG-1013-32058**). The views expressed are those of the authors and not necessarily those of the NHS, the NIHR or the Department of Health.

This manuscript relates to version 4 of the study protocol, dated 03 March 2017.

Competing interests

The authors declare they have no competing interests.

For peer review only

REFERENCES

1. Misono S, Merati AL. Evidence-based practice: evaluation and management of unilateral vocal fold paralysis. *Otolaryngologic clinics of North America* 2012;45(5):1083-108. doi: 10.1016/j.otc.2012.06.011
2. Fang TJ, Li HY, Gliklich RE, et al. Quality of life measures and predictors for adults with unilateral vocal cord paralysis. *The Laryngoscope* 2008;118(10):1837-41. doi: 10.1097/MLG.0b013e31817e7431
3. Smith ME, Ramig LO, Dromey C, et al. Intensive voice treatment in Parkinson disease: laryngostroboscopic findings. *Journal of voice : official journal of the Voice Foundation* 1995;9(4):453-9.
4. Anderson TD, Spiegel JR, Sataloff RT. Thyroplasty revisions: frequency and predictive factors. *Journal of voice : official journal of the Voice Foundation* 2003;17(3):442-8.
5. Maronian N, Waugh P, Robinson L, et al. Electromyographic findings in recurrent laryngeal nerve reinnervation. *The Annals of otology, rhinology, and laryngology* 2003;112(4):314-23.
6. Uloza V, Pribuisiene R, Saferis V. Multidimensional assessment of functional outcomes of medialization thyroplasty. *European archives of oto-rhino-laryngology : official journal of the European Federation of Oto-Rhino-Laryngological Societies* 2005;262(8):616-21. doi: 10.1007/s00405-004-0755-7
7. Lundy DS, Casiano RR, Xue JW, et al. Thyroplasty type I: short- versus long-term results. *Otolaryngology--head and neck surgery : official journal of American Academy of Otolaryngology-Head and Neck Surgery* 2000;122(4):533-6.
8. Wang W, Chen D, Chen S, et al. Laryngeal reinnervation using ansa cervicalis for thyroid surgery-related unilateral vocal fold paralysis: a long-term outcome analysis of 237 cases. *PloS one* 2011;6(4):e19128. doi: 10.1371/journal.pone.0019128
9. Aynehchi BB, McCoul ED, Sundaram K. Systematic review of laryngeal reinnervation techniques. *Otolaryngology--head and neck surgery : official journal of American Academy of Otolaryngology-Head and Neck Surgery* 2010;143(6):749-59. doi: 10.1016/j.otohns.2010.09.031
10. Paniello RC, Edgar JD, Kallogjeri D, et al. Medialization versus reinnervation for unilateral vocal fold paralysis: a multicenter randomized clinical trial. *The Laryngoscope* 2011;121(10):2172-9. doi: 10.1002/lary.21754
11. Donovan JL, Rooshenas L, Jepson M, et al. Optimising recruitment and informed consent in randomised controlled trials: the development and implementation of the Quintet Recruitment Intervention (QRI). *Trials* 2016;17(1):283. doi: 10.1186/s13063-016-1391-4
12. Hirano M. Clinical Examination of Voice. Wien: Springer-Verlag 1981:100.
13. Rosen CA, Lee AS, Osborne J, et al. Development and validation of the voice handicap index-10. *The Laryngoscope* 2004;114(9):1549-56. doi: 10.1097/00005537-200409000-00009
14. Koufman KA. Laryngeal electromyography in clinical practice: indications, techniques, and interpretation. *Phonoscope* 1998;1:57-70.
15. Crumley RL. Teflon versus thyroplasty versus nerve transfer: a comparison. *The Annals of otology, rhinology, and laryngology* 1990;99(10 Pt 1):759-63.
16. Montgomery WW, Blaugrund SM, Varvares MA. Thyroplasty: a new approach. *The Annals of otology, rhinology, and laryngology* 1993;102(8 Pt 1):571-9.
17. EQ-5D http://www.euroqol.org/ [
18. Belafsky PC, Mouadeb DA, Rees CJ, et al. Validity and reliability of the Eating Assessment Tool (EAT-10). *The Annals of otology, rhinology, and laryngology* 2008;117(12):919-24.
19. Wu MC, Chang YC, Wang TG, et al. Evaluating swallowing dysfunction using a 100-ml water swallowing test. *Dysphagia* 2004;19(1):43-7. doi: 10.1007/s00455-003-0030-x
20. Dejonckere PH, Bradley P, Clemente P, et al. A basic protocol for functional assessment of voice pathology, especially for investigating the efficacy of (phonosurgical) treatments and

For peer review only - http://bmjopen.bmj.com/site/about/guidelines.xhtml

evaluating new assessment techniques. Guideline elaborated by the Committee on Phoniatrics of the European Laryngological Society (ELS). *European archives of oto-rhino-laryngology : official journal of the European Federation of Oto-Rhino-Laryngological Societies* 2001;258(2):77-82.
21. Mat Baki M, Wood G, Alston M, et al. Reliability of OperaVOX against Multidimensional Voice Program (MDVP). *Clin Otolaryngol* 2015;40(1):22-8. doi: 10.1111/coa.12313
22. Sidnell J, Stivers T. The Handbook of Conversation Analysis: Wiley-Blackwell 2013.
23. Goetz JP. Ethnographic Research and the Problem of Data Reduction. *Anthr Educ Q* 1981;12(1):51-70.

For peer review only

For peer review only - http://bmjopen.bmj.com/site/about/guidelines.xhtml

Figure 1. Flow diagram of the VOCALIST feasibility study

For peer review only

For peer review only - http://bmjopen.bmj.com/site/about/guidelines.xhtml

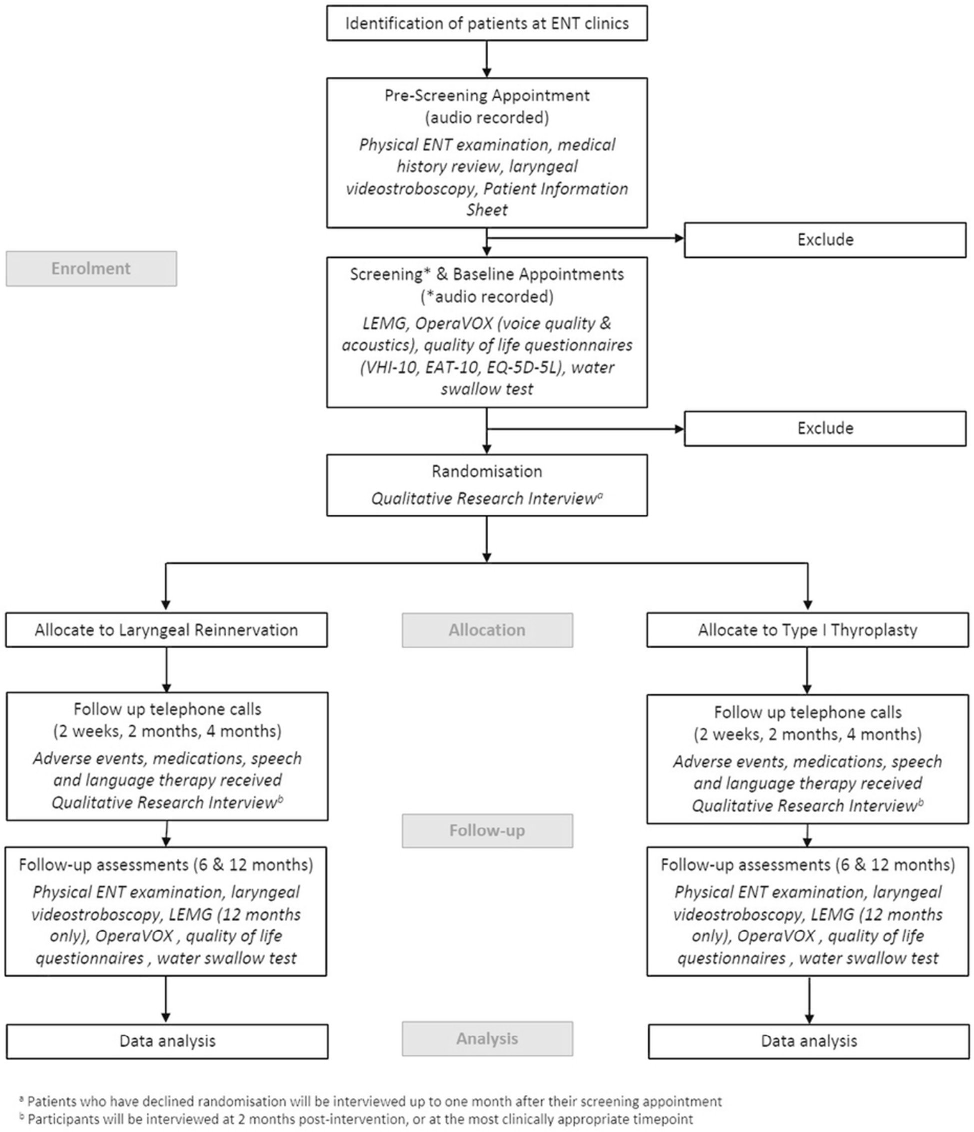

Figure 1. Flow diagram of the VOCALIST feasibility study

80x92mm (300 x 300 DPI)

STANDARD PROTOCOL ITEMS: RECOMMENDATIONS FOR INTERVENTIONAL TRIALS

SPIRIT 2013 Checklist: Recommended items to address in a clinical trial protocol and related documents*

Section/item	Item No	Description	Addressed on page number
**Administrative information**			
Title	1	Descriptive title identifying the study design, population, interventions, and, if applicable, trial acronym	1
Trial registration	2a	Trial identifier and registry name. If not yet registered, name of intended registry	1
	2b	All items from the World Health Organization Trial Registration Data Set	1
Protocol version	3	Date and version identifier	1
Funding	4	Sources and types of financial, material, and other support	1 & 39
Roles and responsibilities	5a	Names, affiliations, and roles of protocol contributors	9
	5b	Name and contact information for the trial sponsor	9
	5c	Role of study sponsor and funders, if any, in study design; collection, management, analysis, and interpretation of data; writing of the report; and the decision to submit the report for publication, including whether they will have ultimate authority over any of these activities	40
	5d	Composition, roles, and responsibilities of the coordinating centre, steering committee, endpoint adjudication committee, data management team, and other individuals or groups overseeing the trial, if applicable (see Item 21a for data monitoring committee)	38

For peer review only

For peer review only - http://bmjopen.bmj.com/site/about/guidelines.xhtml

**Introduction**			
Background and rationale	6a	Description of research question and justification for undertaking the trial, including summary of relevant studies (published and unpublished) examining benefits and harms for each intervention	13-14
	6b	Explanation for choice of comparators	13-14
Objectives	7	Specific objectives or hypotheses	14
Trial design	8	Description of trial design including type of trial (eg, parallel group, crossover, factorial, single group), allocation ratio, and framework (eg, superiority, equivalence, noninferiority, exploratory)	15
**Methods: Participants, interventions, and outcomes**			
Study setting	9	Description of study settings (eg, community clinic, academic hospital) and list of countries where data will be collected. Reference to where list of study sites can be obtained	16
Eligibility criteria	10	Inclusion and exclusion criteria for participants. If applicable, eligibility criteria for study centres and individuals who will perform the interventions (eg, surgeons, psychotherapists)	15-16
Interventions	11a	Interventions for each group with sufficient detail to allow replication, including how and when they will be administered	19, 22-23 & 42-45
	11b	Criteria for discontinuing or modifying allocated interventions for a given trial participant (eg, drug dose change in response to harms, participant request, or improving/worsening disease)	22
	11c	Strategies to improve adherence to intervention protocols, and any procedures for monitoring adherence (eg, drug tablet return, laboratory tests)	39
	11d	Relevant concomitant care and interventions that are permitted or prohibited during the trial	23
Outcomes	12	Primary, secondary, and other outcomes, including the specific measurement variable (eg, systolic blood pressure), analysis metric (eg, change from baseline, final value, time to event), method of aggregation (eg, median, proportion), and time point for each outcome. Explanation of the clinical relevance of chosen efficacy and harm outcomes is strongly recommended	34-35
Participant timeline	13	Time schedule of enrolment, interventions (including any run-ins and washouts), assessments, and visits for participants. A schematic diagram is highly recommended (see Figure)	21

For peer review only - http://bmjopen.bmj.com/site/about/guidelines.xhtml

Sample size	14	Estimated number of participants needed to achieve study objectives and how it was determined, including clinical and statistical assumptions supporting any sample size calculations	35-36
Recruitment	15	Strategies for achieving adequate participant enrolment to reach target sample size	36
**Methods: Assignment of interventions (for controlled trials)**			
Allocation:			
Sequence generation	16a	Method of generating the allocation sequence (eg, computer-generated random numbers), and list of any factors for stratification. To reduce predictability of a random sequence, details of any planned restriction (eg, blocking) should be provided in a separate document that is unavailable to those who enrol participants or assign interventions	38
Allocation concealment mechanism	16b	Mechanism of implementing the allocation sequence (eg, central telephone; sequentially numbered, opaque, sealed envelopes), describing any steps to conceal the sequence until interventions are assigned	38
Implementation	16c	Who will generate the allocation sequence, who will enrol participants, and who will assign participants to interventions	38
Blinding (masking)	17a	Who will be blinded after assignment to interventions (eg, trial participants, care providers, outcome assessors, data analysts), and how	15
	17b	If blinded, circumstances under which unblinding is permissible, and procedure for revealing a participant's allocated intervention during the trial	18
**Methods: Data collection, management, and analysis**			
Data collection methods	18a	Plans for assessment and collection of outcome, baseline, and other trial data, including any related processes to promote data quality (eg, duplicate measurements, training of assessors) and a description of study instruments (eg, questionnaires, laboratory tests) along with their reliability and validity, if known. Reference to where data collection forms can be found, if not in the protocol	30-33
	18b	Plans to promote participant retention and complete follow-up, including list of any outcome data to be collected for participants who discontinue or deviate from intervention protocols	22

For peer review only - http://bmjopen.bmj.com/site/about/guidelines.xhtml

Data management	19	Plans for data entry, coding, security, and storage, including any related processes to promote data quality (eg, double data entry; range checks for data values). Reference to where details of data management procedures can be found, if not in the protocol	33-34
Statistical methods	20a	Statistical methods for analysing primary and secondary outcomes. Reference to where other details of the statistical analysis plan can be found, if not in the protocol	36-38
	20b	Methods for any additional analyses (eg, subgroup and adjusted analyses)	36-38
	20c	Definition of analysis population relating to protocol non-adherence (eg, as randomised analysis), and any statistical methods to handle missing data (eg, multiple imputation)	36-38
**Methods: Monitoring**			
Data monitoring	21a	Composition of data monitoring committee (DMC); summary of its role and reporting structure; statement of whether it is independent from the sponsor and competing interests; and reference to where further details about its charter can be found, if not in the protocol. Alternatively, an explanation of why a DMC is not needed	39
	21b	Description of any interim analyses and stopping guidelines, including who will have access to these interim results and make the final decision to terminate the trial	38
Harms	22	Plans for collecting, assessing, reporting, and managing solicited and spontaneously reported adverse events and other unintended effects of trial interventions or trial conduct	25-29
Auditing	23	Frequency and procedures for auditing trial conduct, if any, and whether the process will be independent from investigators and the sponsor	N/A
**Ethics and dissemination**			
Research ethics approval	24	Plans for seeking research ethics committee/institutional review board (REC/IRB) approval	38-39
Protocol amendments	25	Plans for communicating important protocol modifications (eg, changes to eligibility criteria, outcomes, analyses) to relevant parties (eg, investigators, REC/IRBs, trial participants, trial registries, journals, regulators)	38-39

For peer review only - http://bmjopen.bmj.com/site/about/guidelines.xhtml

Consent or assent	26a	Who will obtain informed consent or assent from potential trial participants or authorised surrogates, and how (see Item 32)	17
	26b	Additional consent provisions for collection and use of participant data and biological specimens in ancillary studies, if applicable	N/A
Confidentiality	27	How personal information about potential and enrolled participants will be collected, shared, and maintained in order to protect confidentiality before, during, and after the trial	30
Declaration of interests	28	Financial and other competing interests for principal investigators for the overall trial and each study site	N/A
Access to data	29	Statement of who will have access to the final trial dataset, and disclosure of contractual agreements that limit such access for investigators	38
Ancillary and post-trial care	30	Provisions, if any, for ancillary and post-trial care, and for compensation to those who suffer harm from trial participation	29
Dissemination policy	31a	Plans for investigators and sponsor to communicate trial results to participants, healthcare professionals, the public, and other relevant groups (eg, via publication, reporting in results databases, or other data sharing arrangements), including any publication restrictions	40
	31b	Authorship eligibility guidelines and any intended use of professional writers	N/A
	31c	Plans, if any, for granting public access to the full protocol, participant-level dataset, and statistical code	N/A
**Appendices**			
Informed consent materials	32	Model consent form and other related documentation given to participants and authorised surrogates	N/A
Biological specimens	33	Plans for collection, laboratory evaluation, and storage of biological specimens for genetic or molecular analysis in the current trial and for future use in ancillary studies, if applicable	N/A

*It is strongly recommended that this checklist be read in conjunction with the SPIRIT 2013 Explanation & Elaboration for important clarification on the items. Amendments to the protocol should be tracked and dated. The SPIRIT checklist is copyrighted by the SPIRIT Group under the Creative Commons "Attribution-NonCommercial-NoDerivs 3.0 Unported" license.

For peer review only - http://bmjopen.bmj.com/site/about/guidelines.xhtml
